# The Effect of Carbodiimide Crosslinkers on Gelatin Hydrogel as a Potential Biomaterial for Gingival Tissue Regeneration

**DOI:** 10.3390/gels10110674

**Published:** 2024-10-22

**Authors:** Dimas Ilham Hutomo, Fathia Agzarine Deandra, Ketherin Ketherin, Elena García-Gareta, Endang Winiati Bachtiar, Lisa Amir, Fatimah Maria Tadjoedin, Adityo Widaryono, Natalina Haerani, Robert Lessang, Yuniarti Soeroso

**Affiliations:** 1 Doctoral Program, Faculty of Dentistry, Universitas Indonesia, Jakarta 10430, Indonesia; dimas.hutomo@ui.ac.id; 2 Department of Periodontology, Faculty of Dentistry, Universitas Indonesia, Jakarta 10430, Indonesia; fatimah.tadjoedin@ui.ac.id (F.M.T.); adityo.widaryono02@ui.ac.id (A.W.); natalina_perio@ui.ac.id (N.H.); robertlessang@gmail.com (R.L.); 3 Postgraduate Program in Periodontology, Department of Periodontology, Faculty of Dentistry, Universitas Indonesia, Jakarta 10430, Indonesia; fathiaagzarinedeandra@gmail.com (F.A.D.); ketherin17@hotmail.com (K.K.); 4 Multiscale in Mechanical and Biological Engineering, Aragon Institute of Engineering Research (I3A), Aragon Institute of Health Research (IIS Aragon), University of Zaragoza, 50009 Zaragoza, Spain; garciage@unizar.es; 5 Division of Biomaterials and Tissue Engineering, UCL Eastman Dental Institute, University College London, London WC1E 6DE, UK; 6 Department of Oral Biology, Faculty of Dentistry, Universitas Indonesia, Jakarta 10430, Indonesia; endang04@ui.ac.id (E.W.B.); lisa.amir@ui.ac.id (L.A.)

**Keywords:** gelatin, hydrogel, EDC/NHS, gingival scaffold

## Abstract

Connective tissue grafts for gingival recession treatment present significant challenges as they require an additional surgical site, leading to increased morbidity, extended operative times, and a more painful postoperative recovery for patients. Gelatin contains the arginine–glycine–aspartic acid (RGD) sequence, which supports cell adhesion and interactions. The development of gelatin hydrogels holds significant promise due to their biocompatibility, ease of customization, and structural resemblance to the extracellular matrix, making them a potential candidate for gingival regeneration. This study aimed to assess the physical and biological properties of crosslinked gelatin hydrogels using EDC/NHS with two crosslinker concentrations (GelCL12 and GelCL24) and compare these to non-crosslinked gelatin. Both groups underwent morphological, rheological, and chemical analysis. Biological assessments were conducted to evaluate human gingival fibroblast (HGF) proliferation, migration, and COL1 expression in response to the scaffolds. The crosslinked gelatin group exhibited greater interconnectivity and better physical characteristics without displaying cytotoxic effects on the cells. FTIR analysis revealed no significant chemical differences between the groups. Notably, the GelCL12 group significantly enhanced HGF migration and upregulated COL1 expression. Overall, GelCL12 met the required physical characteristics and biocompatibility, making it a promising scaffold for future gingival tissue regeneration applications.

## 1. Introduction

Gingiva is a part of periodontal soft tissue that covers the alveolar bone of both the mandibula and maxilla [1]. Since the 1990s, biomaterial scaffolds such as the acellular dermal matrix, collagen matrix, and enamel matrix derivatives have been introduced as autogenous graft substitutions for gingival augmentation [2,3]. The development of biomaterial scaffolds as autogenous graft substitutions for gingival augmentation continues to advance [4]. Scaffolds are three-dimensional porous biomaterials that can promote cell growth [5,6]. Tissue regeneration using scaffolds provides physical and biological support until new tissue forms through cell attachment, proliferation, and differentiation processes, mimicking extracellular matrix deposition [7]. Biomaterial scaffolds for gingival regeneration should meet certain criteria such as biocompatibility, good adaptation and manipulation, space maintenance, clot stabilization, and tissue integration [4]. The stiffness characteristic of available biomaterial scaffolds may lead to poor adaptation on the root surface, limiting their effectiveness [8].

Hydrogels are considered one of the most promising scaffolds for soft tissue development. A hydrogel is a hydrophilic polymer crosslinked through covalent bonds or held together by physical or chemical methods via intramolecular and intermolecular attractions [9,10]. Hydrogels receive attention in tissue engineering because they can hold large amounts of water with structural similarities to some human soft tissues [11,12]. Hydrogels can be designed as scaffolds for soft tissue regeneration because they are highly hydrated and can mimic native soft tissue. The hydrophilic networks of hydrogels allow nutrients and oxygen to diffuse, promoting cell growth [11,13]. The shape adaptability characteristic of hydrogels is beneficial for a minimally invasive approach in oral tissue engineering [1]. The elasticity and soft nature of hydrogels will minimize irritation in the surrounding tissues [14].

Natural polymers, such as gelatin, collagen, hyaluronic acid, chitosan, or alginate, are widely used for hydrogel fabrication due to their good biological properties [15]. Despite collagen being known as the most abundant protein in the extracellular matrix (ECM), its helical structure and amino acid sequences appeared to initiate antigenic and immunogenic responses in vivo [16,17]. Gelatin is a natural polymer obtained from the denaturation and hydrolysis of collagen, resulting in a lack of both tyrosine and tryptophan and very low levels of phenylalanine [18,19,20]. Therefore, the potential for an antigenic response in vivo might be reduced since gelatin has a smaller chance of forming aromatic radicals [21]. As a denaturation product of collagen, the gelatin structure consists of Gly-X-Y sequences (mainly proline and hydroxyproline) and arginine–glutamine–aspartic acid (RGD) sequences, which play a role in integrin-mediated cell adhesion and serve as target sequences of matrix metalloproteinase (MMP), which are suitable for cell remodeling [22,23,24]. There are two types of gelatin based on its extraction method: type A (acid based), which is mainly obtained from pigs, poultry, and fish, and type B (alkaline based) from bovine sources [16,25].

Animal studies by Chen et al. have demonstrated that gelatin-based hydrogels combined with glycidyl methacrylate dextran can enhance the adhesion, proliferation, and osteogenic differentiation of periodontal ligament cells and promote the regeneration of periodontal tissue [26]. Similarly, an in vivo animal study by Xu et al. showed that the injection of chitosan/β-sodium glycerophosphate/gelatin hydrogels containing growth factors effectively supports periodontal bone regeneration [27]. The physical characteristics of gelatin allow the scaffold to form with good flexibility in order to fit deficient formations for use in periodontal regeneration [26]. However, the reversible gelation properties of gelatin and its poor mechanical properties need to be addressed before its use in periodontal regeneration [16,17].

Crosslinking strategies using carbodiimides such as 1-ethyl-3-(3-dimethylaminopropyl)-carbodiimide (EDC) exhibit good biological responses because they are water-soluble zero-length crosslinkers [27,28]. N-hydroxysuccinimide (NHS) is a stabilizer reagent used to enhance crosslinking efficiency with EDC by stabilizing the amine-reactive intermediate [29]. The use of EDC/NHS crosslinkers in gelatin hydrogels has been explored in various contexts. Compared to other crosslinker agents, such as glutaraldehyde, EDC allows the formation of stable covalent bonds without becoming part of a crosslinked gelatin network, which can help avoid cytotoxic effects [30]. EDC can be used to couple polymers containing carboxyl groups and amines [31,32]. The crosslinking process of gelatin with EDC/NHS improves the rigidity, mechanical strength, and thermal stability of hydrogels [31].

Mimicking the physical properties of a scaffold with native tissue architecture at a macroscopic level is important for scaffold implantation and the stimulation of tissue regeneration [7]. If the physical properties of scaffolds are not matched with the tissue, the healing phase can result in poor functionality and a loss of the regenerated tissue [33]. In this study, gelatin was crosslinked with EDC/NHS to make gelatin hydrogels. The effects of crosslinking gelatin hydrogels on physical (morphology, rheology, swelling ratio, and degradation), chemical (molecular groups), and biological characteristics (viability, COL1 expression, and cell migration) were investigated. Human gingival fibroblast (HGF) cells were used to assess the biological characteristics of the gelatin hydrogels in vitro.

## 2. Results and Discussion

### 2.1. Morphology of Hydrogels

**Figure 1:**
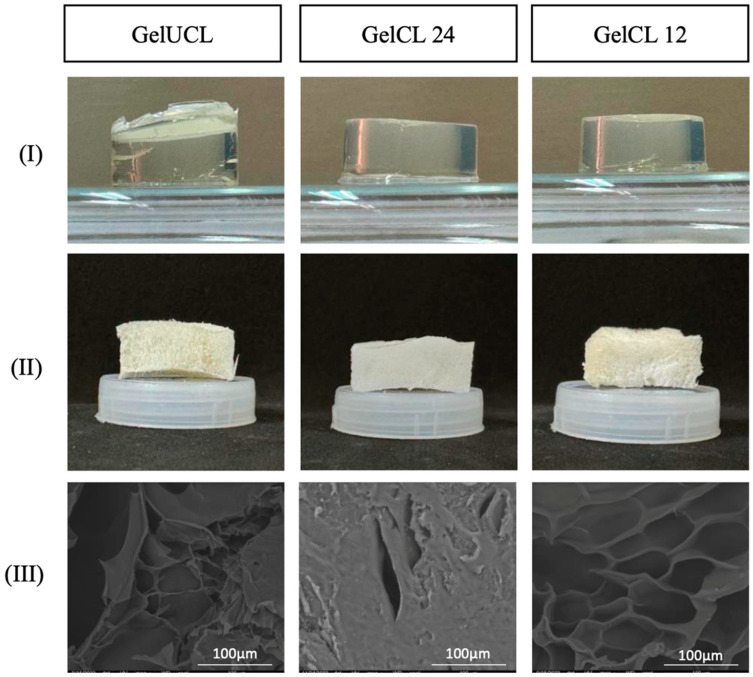
Morphological analysis of gelatin hydrogels: GelUCL, GelCL24, and GelCL12. (**I**) Fresh hydrogels. (**II**) Lyophilized hydrogels. (**III**) SEM analysis of lyophilized hydrogels.

**Figure 2:**
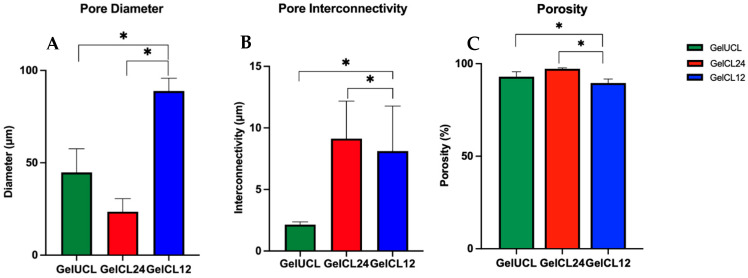
Pore characteristics of gelatin hydrogel groups. (**A**) Comparison of each group’s mean diameter value, with significant differences found between GelUCL and GelCL12 and between GelCL12 and GelCL24. (**B**) Comparison of each group’s mean interconnectivity value, with significant differences found between GelUCL and GelCL12 and between GelCL12 and GelCL24. (**C**) Comparison of each group’s mean porosity value, with significant differences found between GelUCL and GelCL12 and between GelCL12 and GelCL24 (* *p* < 0.05).

Microarchitectural analysis, including pore diameter and interconnectivity, is presented in Figure 1. In this study, we found that GelCL12 showed the largest pore diameter (83.18–96.58 µm), whereas GelCL24 showed the smallest diameter (18.73–31.64 µm), as shown in Figure 2. Interconnectivity is defined as the mean of the distance between adjacent pores. This facilitates cell loading into the scaffold while the inside of the pore wall acts as a vessel for cell attachment and the exchange of nutrients and waste [34,35]. Our study found that GelCL24 and GelCL12 exhibit a significantly higher range of interconnectivity with measurements of 6.12–12.22 µm and 5.57–12.31 µm, respectively. GelUCL had a narrower interconnectivity, with a range from 1.9 to 2.33 µm.

Furthermore, Figure 2C shows the mean porosity of each group. The porosity of GelUCL was measured as 92.97% ± 2.65%. In comparison, the porosity of GelCL24 and GelCL12 was 97.24% ± 0.47% and 89.52% ± 2.19%, respectively. There was a statistically significant difference between GelCL24 and GelCL12 (*p* < 0.05). The data suggest that utilizing the correct amount of the crosslinker makes it possible to attain a scaffold that maintains a somewhat porous and uniform structure in favor of fibroblast growth, while minimizing the possibility of damage to its mechanical strength [1].

### 2.2. Chemical Analysis of Hydrogels

**Figure 3:**
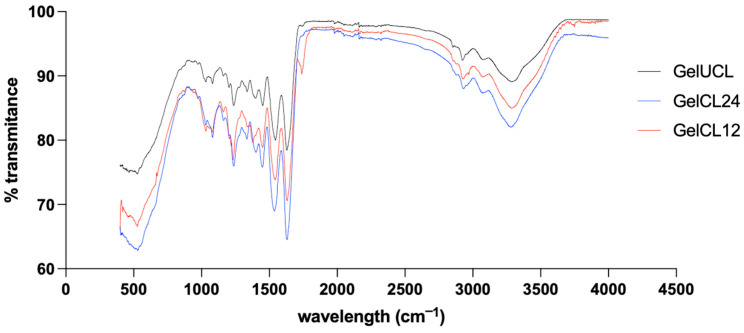
FT-IR spectra of the prepared gelatin hydrogels.

To investigate the influence of the crosslinking agent on the chemical structure of the gelatin hydrogels, FTIR analysis was conducted. The FTIR spectra in Figure 3 indicate that there are similar peptide bond characteristics between the three groups of gelatin hydrogels. The first peak was observed around 3287.00 cm^−1^, confirming the O-H and N-H bonds. The second peak was observed at 2923.77–2926.20 cm^−1^, confirming the -CH_3_ bond. Amide I (1600–1700 cm^−1^) and Amide II (1500–1590 cm^−1^) were found in all of the spectra observed. This indicates that despite the differences in concentration, the structure of the formed crosslinked gelatin hydrogels has not changed significantly due to the crosslinking agents.

### 2.3. Rheological Properties of Hydrogels

**Figure 4:**
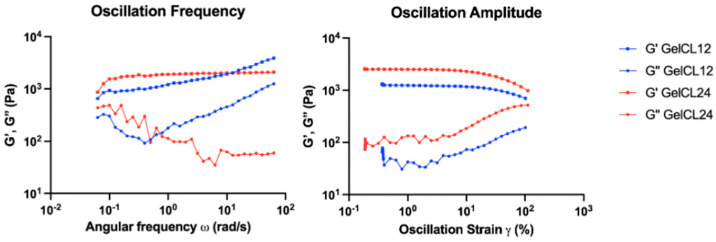
Rheological properties of gelatin hydrogels.

The frequency sweep and strain sweeps were used as rheology measurements in this study and are considered critical for fully characterizing viscoelastic materials. LVR viscosity analysis was carried out to analyze the relationship between oscillation strain and hydrogel shape, which is influenced by the storage modulus (G′) and loss modulus (G″) [36]. The graph in Figure 4 shows that the loss modulus of GelCL12 and GelCL24 was below the storage modulus curve [36], which means that the crosslinked gelatin hydrogels were in a viscoelastic form when a strain of 10^−1^–10^2^ was applied. Sample deformation was seen at a 10% critical strain, marking a non-linear line. A rapid increase in the loss modulus occurred after the critical strain, which marked a change in the properties of the crosslinked gelatin hydrogel. The sample increasingly resembled a fluid-like material, and its ability to store energy elastically decreased. This experimental study found that GelUCL experienced shape degradation when heated to a temperature of 37 °C even before the expected rheological tests were carried out.

### 2.4. Swelling Properties of Hydrogels

**Figure 5:**
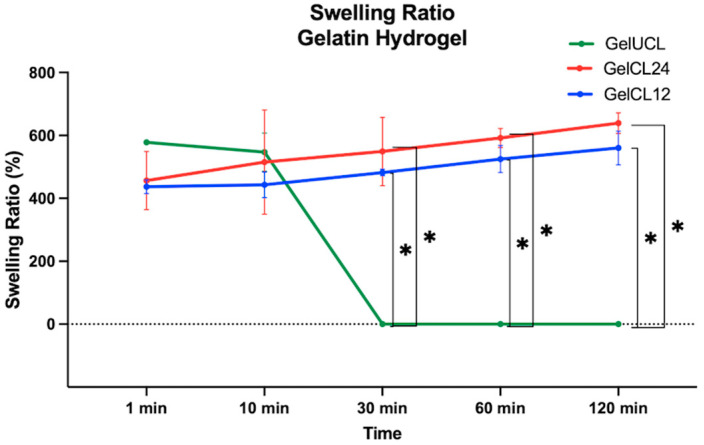
Swelling kinetic curve of GelUCL, GelCL12, and GelCL24 (* *p* < 0.05).

Figure 5 displays the swelling ratio of all samples. Almost all the swelling ratios revealed significant differences across all groups (*p* < 0.05), except for the 1 min and 10 min immersions in PBS. The GelUCL hydrogel demonstrated the highest swelling ratio during the first minute (578.03 ± 7.71), followed by GelCL24 with 456.45% ± 92.49%, and GelCL12 at the lowest with 436.86% ± 21.82%. However, GelUCL no longer held its shape after 30 min or more, so the measurement of its swelling ratio was unfeasible. Overall, GelCL12 had a lower swelling ratio than GelCL24 (*p* < 0.05) due to the more rigid network with the increased amount of the crosslinker agent.

### 2.5. Degradation Rate

**Figure 6:**
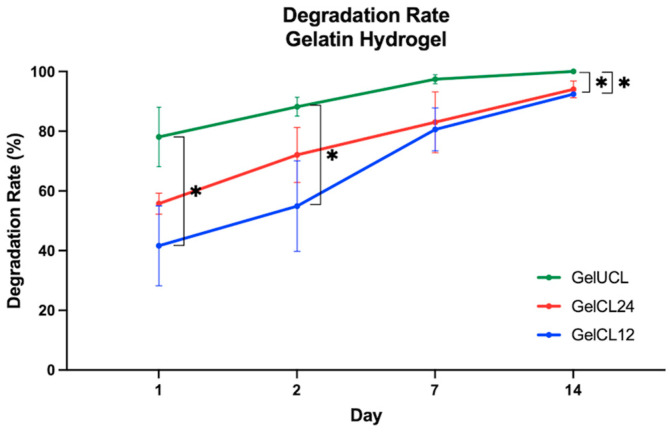
Degradation performance of GelUCL, GelCL12, and GelCL24 (* *p* < 0.05).

The degradation capability of the hydrogels was investigated as a simulation when the scaffolds were implanted in vivo. Figure 6 describes the hydrolytic degradation by soaking the hydrogel in PBS under 37 °C with 5% CO_2_ [37]. Following the first day of immersion in the PBS, GelUCL exhibited a preservation rate of less than 20% of the original dry weight, while Gel CL24 and GelCL12% exhibited preservation rates of over 50% of their original dry weights. These scores are statistically significant differences. On the 7th day, the portion of GelUCL had almost disappeared with 97.41% ± 1.48%. By the 14th day, the remains had completely vanished without leaving any detectable residue. However, samples with EDC/NHS exhibited uncompleted degradation even after 14 days. The degradation rates for GelCL24 and GelCL12 were 94.00% ± 2.80% and 92.43% ± 0.43%, respectively, with no significant difference (*p* > 0.05). This phenomenon suggests that the presence of EDC/NHS may enhance the stability of the hydrogel.

### 2.6. Cell Migration

**Figure 7:**
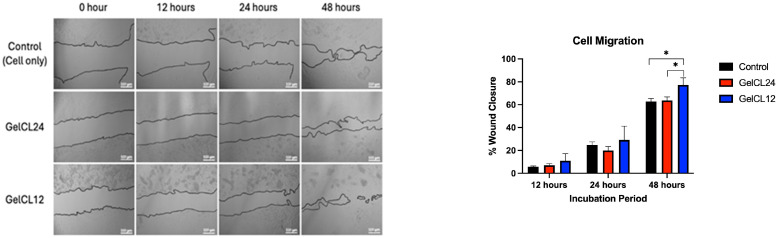
Cell migration assay of HGF monocultures on gelatin hydrogels after 12, 24, and 48 h of incubation. GelCL12 significantly induced HGF migration after 48 h (* *p* < 0.05).

Cell migration is one of the important factors that affect the wound healing process. It is a critical process involved in various physiological and pathological processes, particularly in wound healing. To analyze the ability of the gelatin hydrogels to induce HGF migration, a scratch assay on HGF monocultures was performed and examined at baseline and at 12, 24, and 48 h of incubation by measuring the area as shown in Figure 7. After 72 h of incubation, GelCL12 significantly induced cell migration compared to GelCL24 and the control on the scratch assay (*p* < 0.05).

### 2.7. Cell Viability

**Figure 8:**
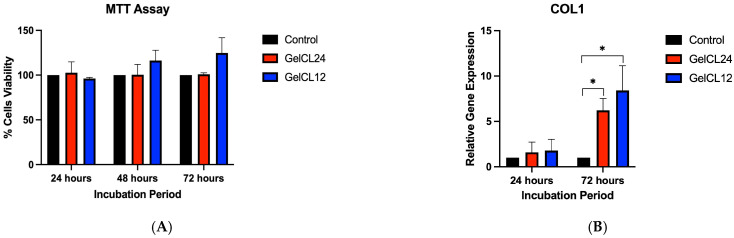
The in vitro behavior of gelatin hydrogels in HGF cells. (**A**) Viability test using the MTT assay on crosslinked gelatin hydrogels after 24, 48, and 72 h of incubation. (**B**) Relative COL1 gene expression showing significant upregulation in GelCL12 after 72 h of incubation (* *p* < 0.05).

HGF viability seeded on gelatin hydrogels was determined after 24, 48, and 72 h of incubation using the MTT assay (Figure 8A). No cytotoxic effects were found on either gelatin hydrogels. There was no significant difference in HGF viability between each gelatin hydrogel and the control. After 48 and 72 h of incubation, there was an increase in HGF viability on GelCL12, while the viability of the cells on GelCL24 remained stagnant.

### 2.8. COL1 Expression

To investigate the effect of gelatin hydrogels on the expression of wound healing-related genes, we used COL1 as a biomarker to analyze the ability of gelatin hydrogels to synthesize type I collagen, an extracellular matrix component. HGFs were seeded into gelatin hydrogels. The proliferation and migration of fibroblast in an area where the scaffold was implanted would result in collagen matrix deposition. After 24 and 72 h, COL1 gene expression was determined using qRT-PCR (Figure 8B). During the 24 h, we found no significance difference in COL1 gene expression. GelCL12 showed significant COL1 gene upregulation at 72 h compared to GelCL24 and the control (*p* < 0.05).

### 2.9. Discussion

Biomaterials act as a substitute for connective tissue grafts that are commonly used in root coverage procedures and soft tissue volume augmentation. The root coverage procedure focuses on the material’s ability to enhance the vertical dimension to compensate for the loss caused by gingival recession. On the other hand, soft tissue volume augmentation aims to increase the tissue volume mainly in the horizontal direction. Ideally, this biomaterial could address both vertical and horizontal tissue regeneration. Various biomaterials have been used and investigated for their effectiveness in gingival regeneration and augmentation. Acellular dermal matrices and collagen matrices have been widely used as alternative biomaterials for gingival tissue grafts [4,38]. Soft tissue engineering is a new approach to repairing damaged organs, including soft tissue. Hydrogels met some criteria as ideal biomaterials to mimic soft tissue and have been developed for this purpose [39].

To make the gelatin hydrogels, we dissolved and stirred gelatin powder in hot water. Gelatin is insoluble in hot water at temperatures above 30 °C [36]. In an attempt to increase the mechanical stability and solubility of the gelatin hydrogels, we used EDC/NHS for the crosslinking strategy. As shown in Figure 1, adding EDC/NHS into gelatin results in the formation of short-range amide bonds between gelatin molecules. EDC activation forms active O-urea, which reacts with amino groups, creating amide links and releasing isourea, which can be easily hydrolyzed. NHS was used to overcome this limitation and form a more stable intermediate prior to amination [40,41]. Carbodiimide activates carboxylic acid residues. EDC/NHS can link amino and carboxylic acid groups within 1 nm of one another [42]. Previously, Goodarzi et al. prepared stable gelatin/EDC/NHS hydrogels with a mass ratio of Gel:EDC:NHS = 12:1:1 [43].

The crosslinked bond influences mechanical properties, swelling ability, and nutritional diffusion across the gel structure. The pores observed in the crosslinked gelatin hydrogel are large with clear interconnectivity, while uncrosslinked gelatin exhibits irregular and non-homogeneous shapes with thin and narrow interconnectivity. Previous studies reported that a pore diameter of 100–135 μm is ideal for facilitating cell attachment; however, an agreed-upon number remains debatable [44]. A pore diameter that is too small prevents cells from migrating to the center of the scaffold, while a pore diameter that is too large reduces the surface area required for cell adhesion [45]. The diffusion process of metabolites, oxygen, and growth factors will pass through the scaffold material so that the open tissue structure will facilitate cell survival and proliferation. The porous structure encourages host–cell infiltration and increases vascularization, providing nutrition to developing tissue [46]. Fibroblasts, which are cells that contribute most to the formation of periodontal connective tissue, were reported to proliferate optimally at a pore diameter of 50–160 μm [1]. Ideally, a scaffold with a porosity ranging from 60% to 90% would be more appropriate for tissue engineering needs. As the level of porosity in the scaffold escalates, a greater amount of empty space is created within the biomaterial, potentially compromising its mechanical strength [47].

FTIR analysis showed that adding EDC/NHS as a crosslinker did not change the chemical bonds inside the gelatin polymers as it is a zero-length crosslinker, confirming Hoon Lee et al.‘s claim [48]. It can be concluded that despite differences in the crosslinking process, the main chemical structure of the gelatin hydrogel remains similar, with hydrogen bonds and key peptide groups playing a central role in its properties. Our study proves that the crosslinked gelatin hydrogel with EDC/NHS has superior thermal stability and structural strength when compared with GelUCL.

Hydrogel is a potential biomaterial for its similarity to the extracellular matrix, which plays an important role in cell development and homeostasis. Hydrogels have unique viscoelastic characteristics with a very low modulus when compared to solid materials. Analysis of mechanical and rheological properties of hydrogels is necessary to understand cell mechanotransduction [47]. The frequency sweep test is carried out by progressively changing the given frequency while keeping the amplitude constant; meanwhile, the strain sweep test provides a fixed frequency but a progressive strain range. The gelatin’s gelation process results from conformational changes (coil-to-helix transition) and the aggregation of protein chains [12]. The new chemical structure created during solution cooling is stabilized by hydrogen bonds. Hydrogen bonds that are easily broken when heated cause gelatin hydrogel to become thermally reversible and have poor mechanical properties.

The hydration of hydrogels relaxes the polymer chain and expansion due to osmotic forces [49]. The swelling ratio is frequently used as a parameter to show the scaffold’s reaction to its surrounding environment [50]. The appearance of swelling can be associated with the formation of hydrogen bonds between the free -OH groups and the molecules that exist in the aqueous solution [51]. These hydrogen bonds facilitate hydrogel gelatin in retaining water within its structure, ultimately resulting in apparent swelling. Numerous studies have reported hydrogels with high swelling rates (>150%), which are advantageous for tissue regeneration and drug delivery [52]. This study found that a higher concentration of crosslinker agents in gelatin hydrogels reduces the swelling ratio, resulting in the enhanced stability and rigidity of the material [53]. This is in line with the research by Pele et al., who reported that the swelling ratio of egg white/gelatin hydrogels in PBS was decreased with a higher glutaraldehyde concentration [54]. As the amount of crosslinker agent increases, the amount of amino and carboxyl groups in the gelatin hydrogel decreases. Hence, the ratio of swelling decreases as well. Moreover, upon exposure to an aqueous solution, gelatin hydrogels also experience a consistency transformation, resembling the structure of rubber. This alteration reduces interfacial tension with other biological fluids, thereby mimicking the characteristics of living tissue [55].

The degradation of the scaffold should ideally synchronize with the formation of the targeted new tissue [1]. In gingival regeneration, 14 days is considered sufficient for facilitating the development of new tissue, infiltration, and the proliferation of cells [37,56]. The degradation rate must not be too slow to avoid infection and nutrient/oxygen deficiencies in the growing tissue [46]. We found that GelCL12 had slower degradation, followed by GelCL24 and GelUCL. A higher crosslinking density restricts the infiltration of water molecules into the internal framework of the scaffold, resulting in more stable hydrogels and a slower degradation rate [57]. This could explain the slower degradation rate observed in GelCL12 when compared to other groups.

An MTT assay was used to investigate the cytotoxic effect of the EDC/NHS concentration in hydrogels. EDC/NHS are carbodiimide agents that are widely used to crosslink gelatin polymers. This study revealed that EDC/NHS had no cytotoxic effect on HGF cells. The cytotoxic effect observed with higher crosslinker concentrations is most likely caused by the residual or unbound crosslinkers within the matrix. This study found higher rates of HGF cell proliferation on GelCL12 with a higher crosslinker concentration. Another study revealed that based on the molar ratio, the highest EDC/NHS concentration, 1 mM, showed superior cell proliferation after 14 days, while the maximum concentration, which had no cytotoxic effect, was 10 mM in the MSC culture [58]. Higher concentrations of EDC and NHS improved collagenase resistance but led to a less favorable surface for cell adherence and proliferation [59].

Collagen type 1 is the most abundant protein found in the gingival extracellular matrix. Gingival fibroblasts expressed the COL1 gene, which is a crucial aspect of gingival regeneration. Research has shown that gingival fibroblasts play a significant role in collagen turnover and synthesis. Gingival fibroblasts can exhibit higher expression levels of collagen genes and proteins, such as collagen type I, MMP-1, and LH2b [60]. The proliferation of gingival fibroblast cells is intricately linked to the expression of the COL1 gene, which encodes collagen type I, a crucial component of the extracellular matrix (ECM) in gingival tissues [61]. This study found that a higher EDC/NHS concentration in gelatin hydrogel promoted higher levels of COL1 gene expression. Previous studies highlighted that gelatin hydrogels influence COL1 gene expression through various pathways, including promoting ECM protein deposition and regulating wound healing gene expression related to tissue repair and regeneration [58,62,63].

Gelatin hydrogels have been extensively studied for their applications in promoting cell behavior, particularly fibroblast migration. Fibroblast migration encompasses a series of events, including the extension of cellular protrusions, stable attachment near the leading edge, forward movement of the cell body, and subsequent release of adhesions at the rear of the cell [64]. Gelatin hydrogels have been linked to increased fibroblast adhesion, spreading, and proliferation. Additionally, the biodegradability of gelatin hydrogels allows host cells such as fibroblasts to migrate through the material, aiding in tissue integration [65].

The results obtained from this study prompted us to analyze the potential of gelatin hydrogels as a substitution for gingival regeneration. Hydrogels can swell by absorbing fluid when they are implanted into tissue, which may be an advantage for enhancing gingival tissue regeneration. For further investigation, the in vivo performance of the crosslinked gelatin hydrogels presented here should be evaluated to test their suitability for gingival regeneration.

## 3. Conclusions

The crosslinking strategy using EDC/NHS modifies the chemical structure of gelatin by forming an amide linkage. This modification enhances the stability of the hydrogels without adversely affecting their biocompatibility or functional properties for use as a scaffold. We found that GelCL12, which has a higher crosslinker degree, demonstrated superior results compared to GelCL24 and GelUCL. In summary, the concentration of EDC/NHS in hydrogels plays a crucial role in modulating cell proliferation. Optimal concentrations can enhance hydrogel stability, resistance to degradation, and cell viability, while excessive concentrations may negatively impact cell adherence and proliferation. Therefore, careful consideration of EDC/NHS concentrations is essential for designing hydrogels for applications requiring cell growth and proliferation. This study was limited by the in vitro nature of the experiments, which may not fully mimic the complexity of the in vivo gingival tissue regeneration process. For further investigation, the in vivo performance of the crosslinked gelatin hydrogels presented here should be evaluated to test their suitability for gingival regeneration. Additionally, incorporating growth factors or cellular components into a hydrogel could further enhance its regenerative properties.

## 4. Materials and Methods

### 4.1. Materials

Gelatin type B powder from bovine skin was purchased from Sigma-Aldrich (St. Louis, MO, USA). N-ethyl-N’-(3-dimethylaminopropyl)carbodiimide liquid (EDC; Mw: 155.24; Sigma Aldrich), N-hydroxysuccinimide powder (NHS; Mw: 115.09; Sigma Aldrich), and phosphate-buffered saline (pH = 7.4) were obtained from Sigma-Aldrich. Distilled water and 95% ethanol were used throughout the experiment.

### 4.2. Preparation of Gelatin Hydrogels

**Figure 9:**
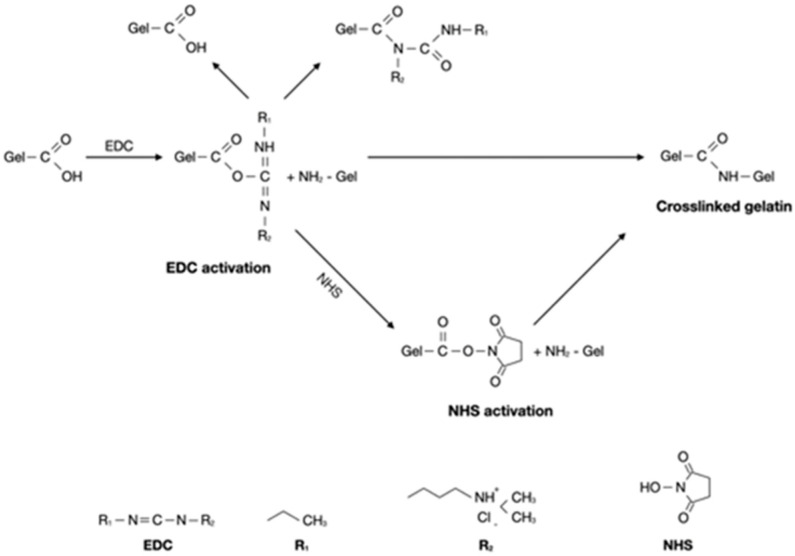
Mechanism of the crosslinking reaction between gelatin and EDC/NHS.

The gelatin hydrogel was prepared by dissolving 1 g of gelatin powder in distilled water at a concentration of 5% *w/v* in a stirrer at 40 °C until it reached a homogenous solution. To the gelatin (Gel) solution, EDC and NHS were added drop by drop with a mass ratio of EDC:NHS:Gel = 1:1:12 (GelCL12) and 1:1:24 (GelCL24). The crosslinking mechanism is explained in Figure 9. The crosslinking process was performed by stirring the mixture in a magnetic stirrer at 4 °C (200–300 rpm for 10 min) until the hydrogel started to form. This method followed the approach described by Goodarzi et al., with some modifications to suit our experimental conditions [45]. The uncrosslinked gelatin (GelUCL) was used as a control.

The solution was then poured into a 10 mL tube for 24 h at 4 °C, forming a hydrogel. To remove the residue of EDC/NHS, the hydrogel underwent a triple rinsing process using distilled water. A two-step freezing was conducted at −20 °C for 7 h, subsequently followed by −80 °C for 24 h. The frozen hydrogels were transferred to a freeze-dryer (Gyrozen Hypercool, Gimpo-si, Republic of Korea) at −110 °C for 24 h to obtain the lyophilized hydrogels. The samples were prepared into 5 × 5 × 5 mm cubes.

### 4.3. Morphology of Hydrogels

The morphology and microstructure of the hydrogels were observed using a scanning electron microscope (SEM) (FEI Inspect F50, Eindhoven, The Netherlands). The lyophilized hydrogel samples were cut and coated with gold for 10 s to provide a conductive surface for electrons prior to SEM. The cross-section of hydrogels was observed at ×1000 magnification. The sizes, interconnectivity, and number of pores were analyzed using ImageJ software (version 1.54j). The liquid displacement method was used to calculate the porosity of the hydrogels.

### 4.4. Chemical Analysis of Hydrogels

The footprint of chemical groups and bonds in the uncrosslinked and crosslinked gelatin hydrogels were analyzed using Fourier transform infrared spectroscopy (FTIR) (Perkin Elmer spectrometer, Shelton, CT, USA). The lyophilized hydrogel samples were ground and placed in a holder in the path of the infrared sources. The spectra of all samples were recorded in the wavenumber range of 4000–400 cm^−1^.

### 4.5. Rheology of Hydrogels

A DHR 1 rheometer (TA Instruments, New Castle, DE, USA) with smart swap geometry was used to measure the rheological properties of the hydrogels. The samples were placed on the plate for gap positioning, immobilized, and trimmed. The oscillatory mode at 37 °C was selected for the rheological tests. We used amplitude and oscillatory sweep data from the linear viscoelastic area (γ = 0.1–1000, ω = 10 Hz) and (ω = 0.01–100 Hz, γ = 100), respectively. The storage modulus (G′) and loss modulus (G″) used to evaluate viscosity were calculated from the linear viscoelastic region (LVR).

### 4.6. Swelling Properties of Hydrogels

Initially, the lyophilized hydrogel samples were cut at 25 mm^3^ and weighed using an analytical balance (Shimadzu AX200, Tokyo, Japan). Subsequently, the samples were soaked in a 5 mL PBS solution at 37 °C and 5% CO_2_. The samples were taken from the PBS, rinsed with distilled water at pre-determined time intervals (1, 10, 30, 60, and 120 min), blotted with filtration paper until no drop was left, and weighted to determine their final weight. The swelling rate (SR) was calculated using the following equation:

$$Swelling\;rate\;\left(\%\right)=\frac{W_{l}-W_{0}}{W_{0}}\times 100\%$$

where *W*_0_ represents the initial weight of the lyophilized hydrogels and *W_l_* is the final weight of the lyophilized hydrogels after finishing the swelling test. All samples were tested in triplicate (*n* = 3).

### 4.7. Degradation Rate

The lyophilized hydrogels were cut into pieces until they reached a similar weight and weighted using an analytical balance (Shimadzu AX200, Tokyo, Japan). The in vitro degradation behavior was measured by immersing the lyophilized hydrogels in a plastic pot containing 5 mL PBS at 37 °C. The precipitates were carefully extracted and rinsed with deionized water at specified time intervals of 1, 2, 7, and 14 days. The final weight was then measured. The PBS solution was replaced every three days.

The degradation rate (DR) was calculated using the following equation:

$$Degradation\;rate\;\left(\%\right)=\frac{W_{0}-W_{t}}{W_{0}}\times 100\%$$

where *W*_0_ is defined as the initial weight of the lyophilized hydrogels and *W_t_* is its final weight after the degradation test. The tests were also performed in triplicate for all samples (*n* = 3).

### 4.8. Cell Culture

Human gingival fibroblast (HGFs) cells (Dainippon Pharmaceutical Co., Ltd., Osaka, Japan) were cultivated in Dulbecco’s modified Eagle’s medium (DMEM) (Solarbio, Beijing, China) containing 10% fetal bovine serum (Biosera, Cholet, France) and 1% AA (Gibco, Waltham, MA, USA). The cells were incubated at 37 °C and 5% CO_2_ in 25 cm^2^ tissue culture flasks (Jetbiofil, Guangzhou, China). Cells in passages 6–8 were used.

### 4.9. Cytotoxicity

A cytotoxicity assay on HGFs was assessed using an MTT assay at 24, 48, and 72 h. The HGF cells were seeded at 10^4^ cells/well in 96-well plates and incubated in a complete medium at 37 °C and 5% CO_2_. After incubating the HGF, the medium was removed and hydrogel elutes in a complete medium were added to the plates and incubated for 24, 48, and 72 h. After incubation, the culture medium from each well was discharged, and 15 μL of the MTT solution was added into each well before incubating at (37 °C, 5% CO_2_) for 3 hrs. An amount of 150 μL of acidified isopropanol solution was added to each well. The OD value was read using an enzyme-linked immunosorbent assay (ELISA) reader (Metertech, Taipei City, Taiwan) at 600 nm wavelength. The viability of HGF was calculated and presented in (%). The MTT assay was performed in quadruplicate in three independent experiments (*n* = 3).

### 4.10. COL1 Expression

Quantitative RT-PCR was used to investigate the expression of the COL1 gene. RNA was reserve transcribed, and relative transcripts for the COL1 gene, glyceraldehyde-3-phosphate dehydrogenase (GAPDH), as a housekeeping gene, were measured using FastStart Universal SYBR Green Master ROX (Roche, Switzerland). Relative gene expression was calculated by normalizing the expression of each gene with GAPDH. The PCR program was set at 95 °C for 5 min, followed by 45 cycles for the amplification phase; each cycle consisted of denaturation for 30 s at 95 °C, annealing for 30 s at 50 °C, and extension for 30 s at 72 °C. The COL1 primer sequences used for PCR amplification were 5′-TCTAGACATGTTCAGCTTTGTGGAC-3′ as its forward primer and 5′-TCTGTACGCAGGTGATTGGTG-3′ for the reverse primer. The forward primer of GAPDH was 5′-GAAGGTCGGAGTCAACGG-3′, while the reverse primer was 5′-GGAAGATGGTGATGGGATT-3′. HGFs were seeded into the 3D gelatin hydrogels, and the COL1 gene expression at 24 and 72 h were determined. Data were analyzed using the efficiency ΔΔCt method. All samples were run in triplicates. All quantitative PCR reactions were performed in quadruplicate in three independent experiments (*n* = 3).

### 4.11. Cell Migration

Cell migration behavior was performed in vitro using the scratch assay technique by seeding HGF cells at a density of 10^4^ cells/well in a 24-well plate and incubated at 37 °C under 5% CO_2._ After reaching 80% confluency, the medium was changed to a low serum medium (DMEM + 0.2% FBS + 1% AA) and incubated for 24 h. A 20 μl pipette tip was used to scratch the monolayer mechanically. PBS was used to gently rinse the cell debris. The cells were captured using an inverted optical microscope (Axiovert 40 CFL, Zeiss, Germany) with ×4 magnification. After that, the hydrogel elusion was added to the well plate, and the cells were incubated at 37 °C under 5% CO_2_. Three different time points (12, 24, and 48 h) were used to calculate the wound closure. The gap size at each time point was measured using ImageJ and calculated using the following equation:

$$Wound\;closure\;\left(\%\right)=\frac{A_{t=0}-A_{t=∆t}}{A_{t=0}}\times 100\%$$

where $A_{t=0}$ is the initial wound area and $A_{t=∆t}$ is the wound area after n hours of the initial scratch, both in μm^2^. Cell migration was performed in quadruplicate in three individual experiments (*n* = 3).

### 4.12. Statistical Analysis

The data were reported as mean ± standard deviation (SD). Statistical analysis was calculated using ANOVA with Tukey’s post hoc test for multiple comparisons using SPSS 26.0. One sample *t*-test compared the hydrogels’ cell viability and COL1 expression to the control. A *p-*value < 0.05 was considered statistically significant for all comparisons.

## Data Availability

The original contributions presented in the study are included in the article, further inquiries can be directed to the corresponding author.
